# Identification of an EMT‐related gene‐based prognostic signature in osteosarcoma

**DOI:** 10.1002/cam4.5942

**Published:** 2023-04-27

**Authors:** Haoli Gong, Ye Tao, Sheng Xiao, Xin Li, Ke Fang, Jie Wen, Ming Zeng, Yiheng Liu, Yang Chen

**Affiliations:** ^1^ Department of Orthopedics Hunan Provincial People's Hospital (The First‐Affiliated Hospital of Hunan Normal University) Changsha China; ^2^ Department of Radiology The Third Xiangya Hospital Central South University Hunan Changsha China; ^3^ Department of Orthopedics Haikou Affiliated Hospital of Central South University Xiangya School of Medicine Hai kou China; ^4^ Department of Orthopaedics The Third Xiangya Hospital Central South University Changsha China

**Keywords:** CDK3, EMT, immune infiltration, osteosarcoma, prognosis

## Abstract

**Background:**

The correlation between epithelial‐mesenchymal transition (EMT) and osteosarcoma (OS) has been widely reported. Integration of the EMT‐related genes to predict the prognosis is significant for investigating the mechanism of EMT in OS. Here, we aimed to construct a prognostic EMT‐related gene signature for OS.

**Methods:**

Transcriptomic and survival data of OS patients were downloaded from Therapeutically Applicable Research to Generate Effective Treatments (TARGET) and Gene Expression Omnibus (GEO). We performed univariate Cox regression, least absolute shrinkage and selection operator (LASSO) regression, and stepwise multivariate Cox regression analysis to construct EMT‐related gene signatures. Kaplan–Meier analysis and time‐dependent receiver operating characteristic (ROC) were applied to evaluate its predictive performance. GSVA, ssGSEA, ESTIMATE, and scRNA‐seq were performed to investigate the tumor microenvironment, and the correlation between IC50 of drugs and ERG score was investigated. Furthermore, Edu and transwell experiments were conducted to assess the malignancy of OS cells.

**Results:**

We constructed a novel EMT‐related gene signature (including CDK3, MYC, UHRF2, STC2, COL5A2, MMD, and EHMT2) for outcome prediction of OS. According to the signature, patients stratified into high‐ and low‐ERG‐score groups exhibited significantly different prognoses. ROC curves and Kaplan–Meier analysis revealed a promising performance of the signature with external validation. GSVA, ssGSEA, ESTIMATE algorithm, and scRNA‐seq excavated EMT‐related pathways and suggested the correlation between ERG score and immune activation. Notably, the pivotal gene CDK3 was upregulated in OS tissue and positively related to OS cell proliferation and migration.

**Conclusion:**

Our EMT‐related gene signature might reference OS risk stratification and guide clinical strategies as an independent prognostic factor in OS.

## Graphical abstract

Flowchart of study design.

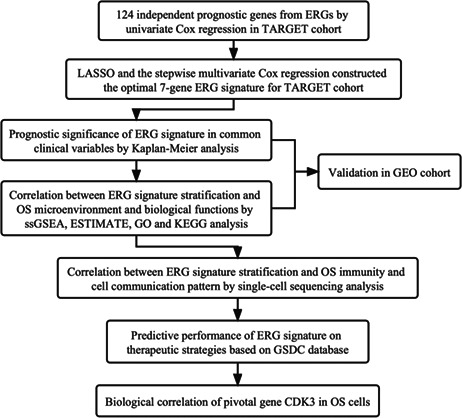

## 1 INTRODUCTION

Osteosarcoma (OS) is a primary malignant bone tumor of mesenchymal origin that frequently occurs in children, adolescents, and young adults. OS tends to arise in the metaphysis of long bones and most commonly affects the distal femur, proximal tibia, and proximal humerus.^1^ Approximately 15%–20% of OS patients have metastatic lesions detected in clinical diagnosis, and over 85% of metastasis affects the lungs, whereas bone is the second most common site of distant metastasis.^2^ The current treatment strategy of neoadjuvant and adjuvant chemotherapy combined with surgical resection has improved the overall survival of nonmetastatic‐OS patients to 70%. However, the five‐year survival rate of patients with distant metastasis, recurrence, or chemo‐resistance remains approximately 20%.^3^, ^4^ Consequently, complex immunogenic mechanisms and intrinsic cellular heterogeneity significantly affect a patient's outcome after therapeutic intervention.^5^ Therefore, the unique tumor microenvironment (TME) may be an environment beneficial for OS cells to develop and metastasize, which has not yet been studied in depth.

Epithelial‐mesenchymal transition (EMT) is a biological process in which polarized epithelial cells change their epithelial phenotype and transform into cells with mesenchymal characteristics, such as enhanced migratory capacity, invasiveness, and elevated resistance to apoptosis.^4^, ^6^ In specific cancer populations, cancer cells acquire stem‐like features, reduced polarity, weakened intercellular adhesion, and present high metastatic potential through EMT‐related pathways.^7^ Increasing evidence has revealed that the EMT process correlated with multiple factors, including transcriptional factors like Snail, Slug, Twist, ZEB, and activation of certain signaling pathways, such as Wnt/β‐catenin, TGF‐β/Samd, and Hedgehog signaling pathway.^8^, ^9^, ^10^ Moreover, EMT is associated with initiation, progression, and lung‐metastasis in OS,^8^, ^11^, ^12^ and EMT affects the drug resistance of malignant cells.^13^, ^14^ This evidence suggests that the EMT signature could be a prognostic factor of OS. However, the EMT and OS microenvironment association has not been sufficiently reported.

The current risk stratification applied in OS primarily focuses on clinical features (size and site of the tumor, patient age, and response to chemotherapy).^15^, ^16^ However, genomic factors have not yet been used in standard clinical applications. Hence, we attempted to construct a prognostic EMT‐related gene (ERG) risk signature for OS to further verify the effect of key genes on OS cells, which may lay the groundwork for further studies or personalized treatment.

## 2 MATERIALS AND METHODS

### 2.1 Data collection

We extracted TARGET‐OS counts data of 84 OS patients with analyzable clinical information from the UCSC Xena website (https://xenabrowser.net/) as a training cohort and RNA‐seq of 53 OS patients from GSE21257^17^ in the GEO database (https://www.ncbi.nlm.nih.gov/geo) was used as the validation cohort. The clinical characteristics of the OS patients are displayed in Table S1. Counts matrices were standardized with “DEseq2” R package. Single‐cell RNA‐seq data containing primary and metastatic OS lesions were collected from GSE152048^18^ in the GEO database. ERGs were attained from the Epithelial‐Mesenchymal Transition Gene Database (http://dbemt.bioinfo‐minzhao.org/download.cgi), and the Molecular Signatures Database v7.5.1 (https://www.gsea‐msigdb.org/gsea/msigdb/index.jsp), and all ERGs are listed in Table S2.

### 2.2 Construction and validation of the EMT‐Related gene signature

The TARGET cohort was used to construct the ERG risk signature, while the GEO cohort was used for validation. Univariate Cox regression analysis was performed to identify 124 independent prognosis‐related ERGs (*p* < 0.05). Then, we used the least absolute shrinkage and selection operator (LASSO) algorithm to filter out 13 ERGs that met the minimum lambda value. Finally, stepwise multivariate Cox regression analysis identified seven ERGs with optimal collinearity to construct the risk signature. The risk score of every OS patient was calculated using the following formula: $\mathrm{ERG}\;\text{score}=\sum_{i=1}^{n}\text{Expi}*\text{Coei}$, where the *n* is the total number of ERGs, the Exp refers to the relative expression level of each ERGs transformed from our expression matrix, and the Coe is the regression coefficient. Hazard ratios (HRs) were calculated to distinguish between risky (HR > 1) and protective factors (HR < 1). Kaplan–Meier analysis and the log‐rank method were performed with “survival” and “survminer” packages to evaluate the prognostic significance of ERG score. *p* < 0.05 was considered statistically significant. The optimum cut‐off point was calculated using “surv_cutpoint” function to divide OS patients into high‐ERG‐score and low‐ERG‐score groups. Receiver operating characteristic (ROC) analyses were performed using “timeROC” R package to assess the specificity and sensitivity of ERG risk signature.

### 2.3 Correlation analysis between TME and constructed signature

Single‐sample gene‐set enrichment analysis (ssGSEA) was performed to evaluate the relative expression abundance of 28 specific immune cells in two ERG‐score groups. Gene sets of these infiltrating immune cells cover activated B cells, CD4+ T cells, CD8+ T cells, macrophages, NK cells, and so on, collected from previous research.^19^, ^20^ The boxplot was developed using the “ggpubr” R package. Estimated Stromal and Immune cells in Malignant Tumor tissues using Expression (ESTIMATE) was subsequently applied to estimate the relative infiltration level of stromal cells and immune cells. The ESTIMATE score was calculated from stromal and immune scores to evaluate tumor purity.^21^ The half violin diagram was generated using “Rmisc” and “ggunchained” R packages, and scatter diagrams were created by the “ggplot2” R package.

### 2.4 Functional annotation and Gene Set Variation Analysis (GSVA)

Differentially expressed genes (DEGs) between the high‐ERG‐score group and the low‐ERG‐score group were obtained using the “limma” R package. DEGs were visualized in the volcano diagram and heatmap by “EnhancedVolcano” and “pheatmap” R packages, respectively. Based on these DEGs, gene ontology (GO) enrichment and kyoto encyclopedia of genes and genomes (KEGG) pathway analysis were performed with “clusterProfiler” and “org.Hs.eg.db” R packages. Histograms were created using the “ggplot2” R package. Moreover, we performed GSVA enrichment analysis with the “GSVA” R package based on two gene sets, “c2.cp.kegg.v7.4.symbols” and “c5.go.bp.v7.4.symbols”, both of which were extracted from MSigDB database.

### 2.5 Single‐cell RNA sequencing analysis

Single‐cell RNA expression matrix involving primary and metastatic OS lesions was processed using the “Seurat” R package. Initially, we performed “NormalizedData” function to normalize the expression matrix. Then, the “FindVariableFeatures” function was applied to identify the 1000 most variable genes. After “RunPCA,” a K‐nearest neighbor graph was conducted using “FindNeighbors,” followed by cell combination via the “FindClusters” function. Uniform manifold approximation and projection for dimension reduction (UMAP)^22^ was subsequently used for visualization. Then, the “Single R" R package was performed to annotate cells, and all feature genes for annotating the designated cell categories were extracted from previous research.^18^ The identified risk cell cluster's DEGs were calculated with the “FindMarkers” function. Furthermore, the pseudotime trajectory analysis was conducted with the R package “monocle”. The R package “iTalk” was also performed to explore the cell communication pattern.

### 2.6 Therapeutic prediction potential of ERG signature

Using the public database Genomics of Drug Sensitivity in Cancer (GDSC, http://www.cancerrxgene.org/),^23^ we extracted the expression matrix and response to chemotherapy and small‐molecule drugs of over 1000 cancer cell lines. Following extracting, the ERG scores of cell lines were calculated, and the half‐maximal inhibitory concentration (IC50) was estimated to represent the drug response.^24^ Then spearman method was applied to evaluate the correlation (Cor) between ERG scores and IC50. *p* < 0.05 were considered statistically significant.

### 2.7 Immunohistochemistry (IHC)

Three pairs of OS and para‐carcinoma tissue from three patients with OS were fixed with formalin, embedded in paraffin (all post‐chemotherapy), and then made into 5 μm paraffin sections. IHC was performed using the Mouse/rabbit‐enhanced polymer method detection system (ZSGB‐BIO, PV‐9000, China). Moreso, the slides were deparaffinized and rehydrated with xylene and gradient‐concentration ethyl alcohol, and antigen retrieval was performed with sodium citrate at 95°C. Subsequently, the slides were blocked with an endogenous peroxidase blocker for 10 min at room temperature. Samples were incubated with primary antibodies against CDK3 (Proteintech) overnight at 4°C, followed by reaction enhancer for 20 min at 37°C, and enhanced enzyme‐conjugated sheep anti‐mouse/rabbit IgG polymer for 20 min at 37°C. Furthermore, the slides were stained with 3, 30‐diaminobenzidine tetrahydrochloride (DAB) and counterstained with hematoxylin. Images were captured with a magnification of 20x.

### 2.8 Cells culture

Procell Life Science&Technology Co., Ltd provided two OS cell lines, U2OS and MNNG/HOS. U2OS was cultured in McCoy's 5A (Procell), and MNNG/HOS was cultured in MEM (Procell). Media for both cell lines were supplemented with 1% penicillin–streptomycin solution (Biosharp) and 10% fetal bovine serum (Gibco). Cells were cultured at 37°C with 5% CO2 and saturated humidity. The cell medium was refreshed every 24 h, and cells were digested with trypsin–EDTA (Gibco) for passage cultivation.

### 2.9 Cell transfection and real‐time quantitative polymerase chain reaction (RT‐qPCR)

Human CDK3 siRNA (siCDK3) and nonspecific control siRNA (siNC) were synthesized and provided by JTSBio. According to the manufacturer's protocol, OS cells were seeded in a six‐well plate at 2.5 × 10^5^ cells per well and transfected with jetPRIME transfection reagent (Polyplus, France). We extracted the total RNA of these cells with Rnafast200 (Fastagen, Japan) 48 h after transfection. HiScript II Q RT SuperMix (Vazyme, China) synthesized cDNA for RT‐qPCR. The PCR amplification system was conducted with ChamQ Universal SYBR qPCR Master Mix (Vazyme, China) based on the manufacturer's introduction. The PCR reaction was performed according to the following steps: initial denaturation at 95°C for 30 s, 1 repetition; denaturation at 95°C for 10 s, 60°C for 30 s, 40 repetitions; dissolution curve at 95°C for 15 s, 60°C for 60s, 95°C for 15 s, 1 repetition. Gene expression was normalized based on GAPDH and calculated by the lg2–△△Ct method. The sequences of siRNA and primers are listed in Table S3.

### 2.10 EdU incorporation assay

The Click‐iT Plus EdU Alexa Fluor 488 Imaging Kit (Invitrogen) was used to identify proliferating cells, and we stained all cell nuclei with Hoechst (Invitrogen). Under an inverted fluorescence microscope, proliferating cells and cell nuclei appeared red and blue, respectively.

### 2.11 Cell migration assay

The OS cell migration assay used a Transwell chamber (Corning) with polycarbonic membranes (6.5 mm in diameter and 8 μm pore size). Cells were seeded into the upper chamber with serum‐free media at a density of 2.5 × 10^5^ cells/mL (200 μL/chamber), while 750 μL of complete media was added in the lower chamber. Non‐migrated cells were removed using a cotton swab after 48 h incubation in a 37 °C thermostatic incubator. Cells adhered to the lower surface of the membranes were stained purple using 0.1% crystal violet. Finally, cells in five random fields under 200 × magnification per well were counted as *n* = 1 in triplicate.

### 2.12 Statistical analysis

R version 4.0.3 (https://www.r‐project.org/) and GraphPad Prism version 9.0.0 were used for bioinformatic statistical analyses and visualization. A Wilcoxon test was performed to compare the continuous variables of abnormal distribution. Spearman analysis was used to evaluate the correlation among continuous variables, and experimental data were compared using mean ± SD unless otherwise noted. *p.adjust* <0.05 was regarded as statistically significant.

## 3 RESULTS

### 3.1 Identification of prognostic ERGs and construction of an ERG‐based risk signature

**FIGURE 1:**
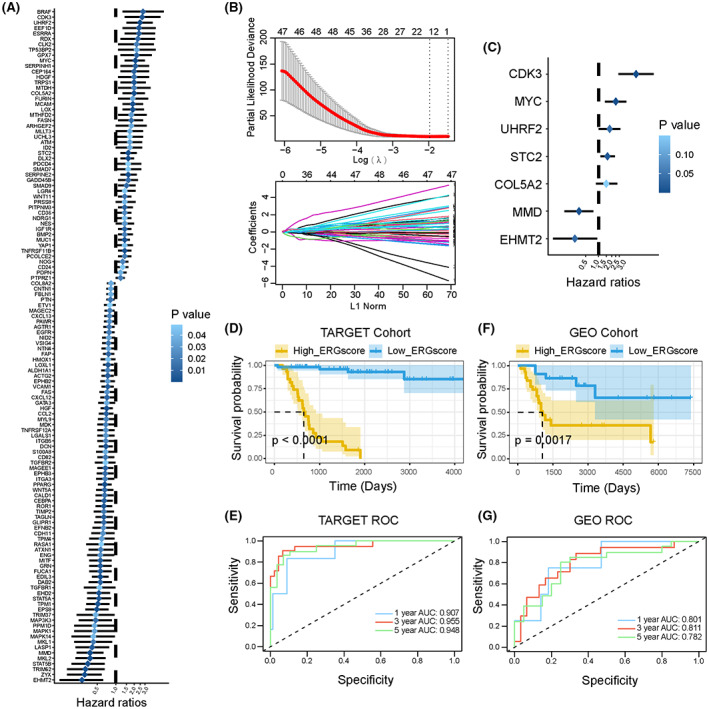
Construction of a survival‐related signature based on EMT‐related genes (ERGs). (A) The forest plot of 124 independent prognostic genes in OS patients (*p* < 0.05). Hazard ratios (HR) and *p*‐value were calculated by univariate Cox regression analysis. The deeper the blue, the greater the significance. (B) LASSO algorithm confirmed minimum lambda value. (C) The forest plot of seven EMT‐related genes in the optimal prognostic model constructed by stepwise multivariate Cox regression analysis. (D) Kaplan–Meier survival curves for OS patients with high‐ and low‐ERG scores in the TARGET cohort. (E) The ROC curves for 1‐, 3‐, and 5‐year survival of constructed ERG signature in the TARGET cohort. (F) Kaplan–Meier survival curves for OS patients with high‐ and low‐ERG scores in the GEO cohort. (G) The ROC curves for 1‐, 3‐, and 5‐year survival of constructed ERG signature in the GEO cohort.

Based on the TARGET training cohort, we screened 124 independent prognostic genes out of 1024 attained ERGs using univariate Cox regression (*p* < 0.05) (Figure 1A). The LASSO algorithm identified 13 ERGs that fulfilled the minimum lambda value from 124 ERGs (Figure 1B). Based on the above LASSO result, we adopted stepwise multivariate Cox regression analysis to generate an optimal prognostic ERG signature containing seven ERGs (Figure 1C): CDK3, MYC, UHRF2, STC2, COL5A2, MMD, and EHMT2. According to Kaplan–Meier analysis, these ERGs were verified to be independent prognostic factors for OS patients (Figure S1A–G). The ERG score of every patient was calculated using the following specific formula: $\begin{array}{c} \mathrm{ERG}\;\text{score}=\left(2.0065\times \text{ExpCDK}3\right)+\left(0.9253\times \text{ExpMYC}\right)\\ +\left(0.6018\times \text{ExpUHRF}2\right)+\left(0.4926\times \text{ExpSTC}2\right)\\ +\left(0.418\times \text{ExpCOL}5A2\right)+\left(-1.0587\times \text{ExpMMD}\right)\\ +\left(-1.2564\times \text{ExpEHMT}2\right) \end{array}$

The optimum cut‐off point for determining the patients' classification of survival subgroups was calculated using the “surv_cutpoint” algorithm of the “survival” R package.^25^ Based on the optimal cut‐off point, the Kaplan–Meier analysis indicated that the low‐ERG‐score group exhibited better survival outcomes compared with the high‐ERG‐score group (*p* < 0.0001) (Figure 1D). We assessed the predictive accuracy of the ERG signature at 1, 3, and 5 years using ROC curve analysis. The area under the ROC curve (AUC) was 0.907, 0.955, and 0.948 at 1, 3, and 5 years, respectively (Figure 1E). Next, we evaluated the predictive applicability and stability of the ERG signature according to an external validation cohort, GSE21257. The ERG score of each patient in the validation cohort was calculated using the formula described above. Consistent with the training cohort, the Kaplan–Meier method showed that patients in the high‐ERG‐score group had shorter overall survival (*p* = 0.0017) (Figure 1F), and the AUC values for ERG signature at 1, 3, and 5 years were 0.801, 0.811, and 0.782, respectively (Figure 1G). Therefore, we have demonstrated that the ERG signature exhibited a credible predictive capacity for OS patients.

### 3.2 Prognostic significance of ERG signature in common clinical variables

**FIGURE 2:**
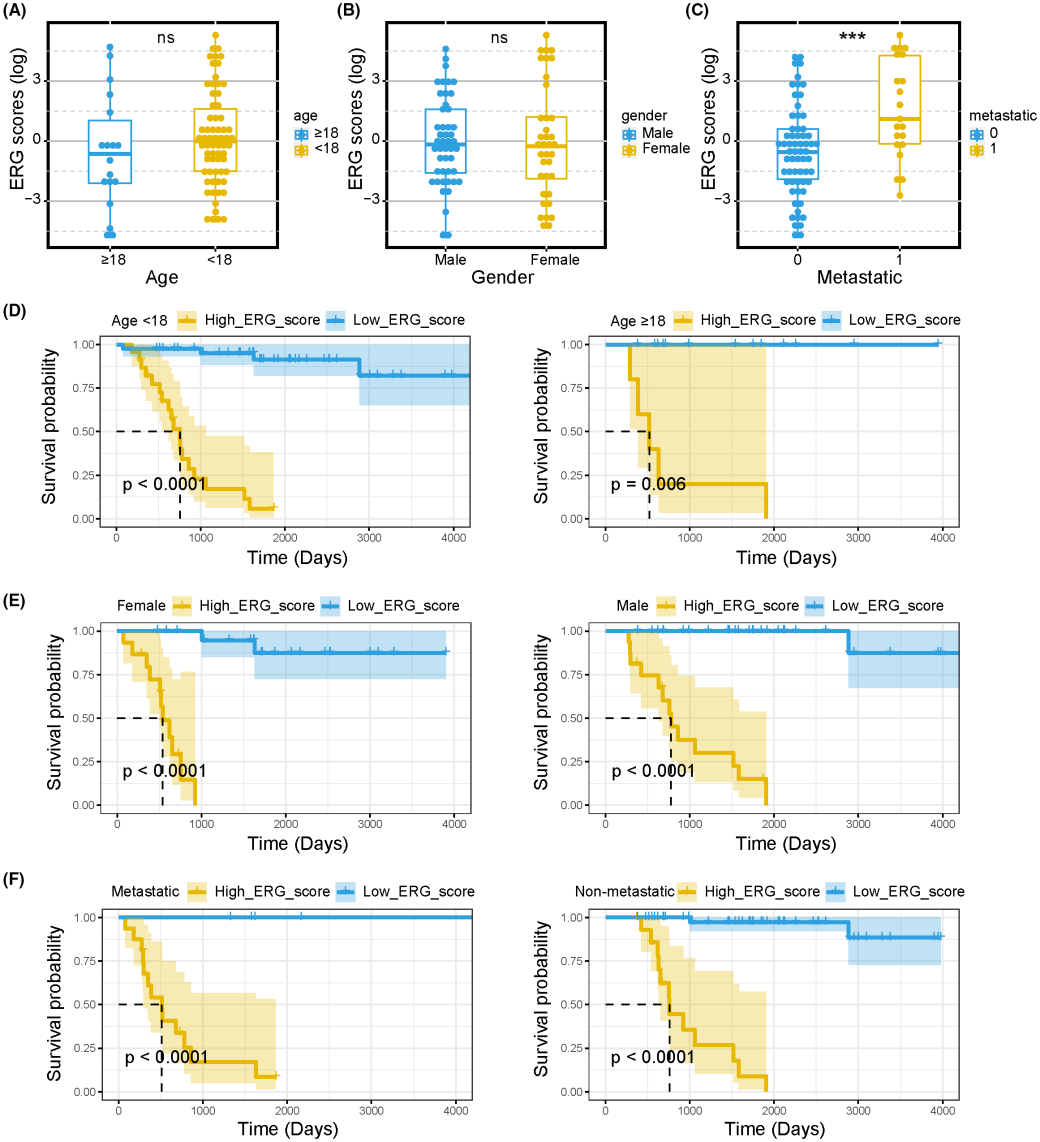
Subgroup analyses of the prognostic value of ERG signature in TARGET‐OS patients. Boxplots of the ERG score in OS stratified by age (A), gender (B), and metastasis (C). Kaplan–Meier survival curves of OS patients with different ages (D), genders (E), and metastatic states (F). Kruskal test *p < 0.05; **p < 0.01; ***p < 0.001; ****p < 0.0001; ns, no significance.

Three common clinical variables, age, gender, and metastasis, were extracted to evaluate the predictive performance of the ERG signature in the TARGET cohort. The results showed that the distribution of ERG scores was independent of age and gender (Figure 2A,B). However, ERG scores in metastatic samples were significantly higher than those in non‐metastatic samples (Figure 2C). Under different clinical stratification, the Kaplan–Meier analysis was applied to compare the overall survival of OS patients in high‐ERG‐score and low‐ERG‐score subgroups. The results indicated that the high‐ERG‐score group had a worse prognosis in any clinical case variable stratification (Figure 2D–F). Similar outcomes were observed in the validation cohort. The ERG scores of metastatic patients were statistically higher than those of non‐metastatic ones (Figure S2A). The distribution of ERG scores was not significantly different regarding age, gender, and Huvos grade stratifications (Figure S2B–D). We identified that the high‐ERG‐score group had worse survival outcomes under different clinical stratification (Figure S2E–H). These results suggest that the prognostic ERG signature could accurately filter out OS patients with poorer or better prognoses without considering the above clinical variables.

### 3.3 ERG signature stratification correlated with OS microenvironment and biological functions

**FIGURE 3:**
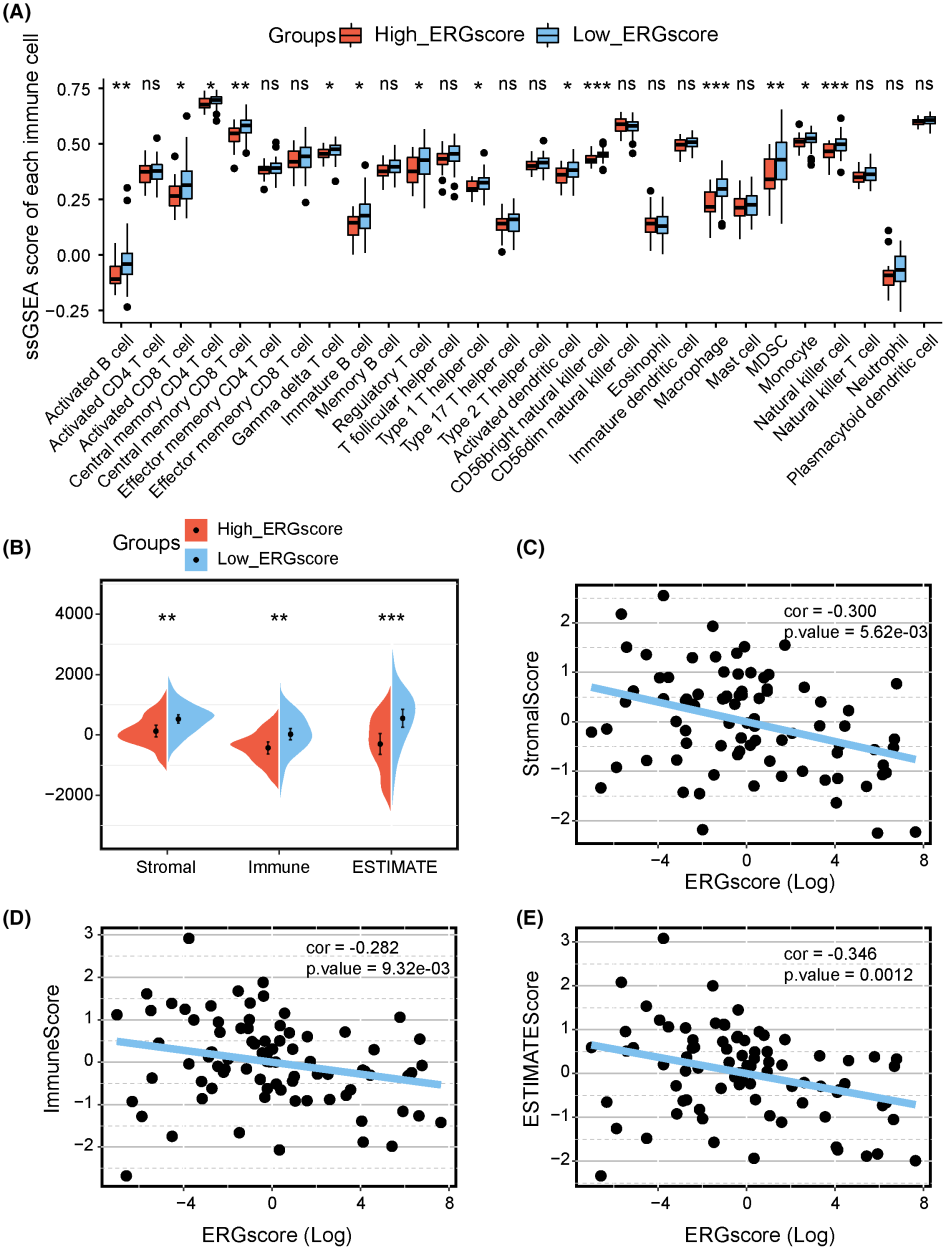
Associations of immune cell infiltration, ESTIMATE scores, and the ERG signature stratification. (A) Comparison of 28 immune cells expression abundance in high‐ and low‐ERG‐score groups based on ssGSEA analysis. (B) Comparison of Stromal, immune, and ESTIMATE scores in high‐ and low‐ERG‐score groups. Scatter plots show the spearman correlation between ERG score and stromal score (C), immune score (D), and ESTIMATE score (E). Kruskal test *p < 0.05; **p < 0.01; ***p < 0.001; ****p < 0.0001; ns, no significance.

Expression abundance of multiple tumor immune‐associated cells was compared in the high‐ versus low‐ERG‐score groups. The ssGSEA analysis demonstrated that in the high‐ERG‐score group, the abundance of activated B cells, CD8+ T cells, central memory CD4 T cells, central memory CD8 T cells, gamma delta T cells, immature B cells, regulatory T cells, type 1 T helper cells, activated dendritic cell, CD56+ NK cells, macrophages, MDSCs, monocytes, and NK cells were markedly decreased (Figure 3A). From the ESTIMATE analysis, we found that the low‐ERG‐score group had a higher stromal score, immune score, and ESTIMATE score (Figure 3B). Furthermore, Spearman analysis results showed that the ERG score was negatively correlated with the stromal score (Figure 3C), immune score (Figure 3D), and ESTIMATE score (Figure 3E).

**FIGURE 4:**
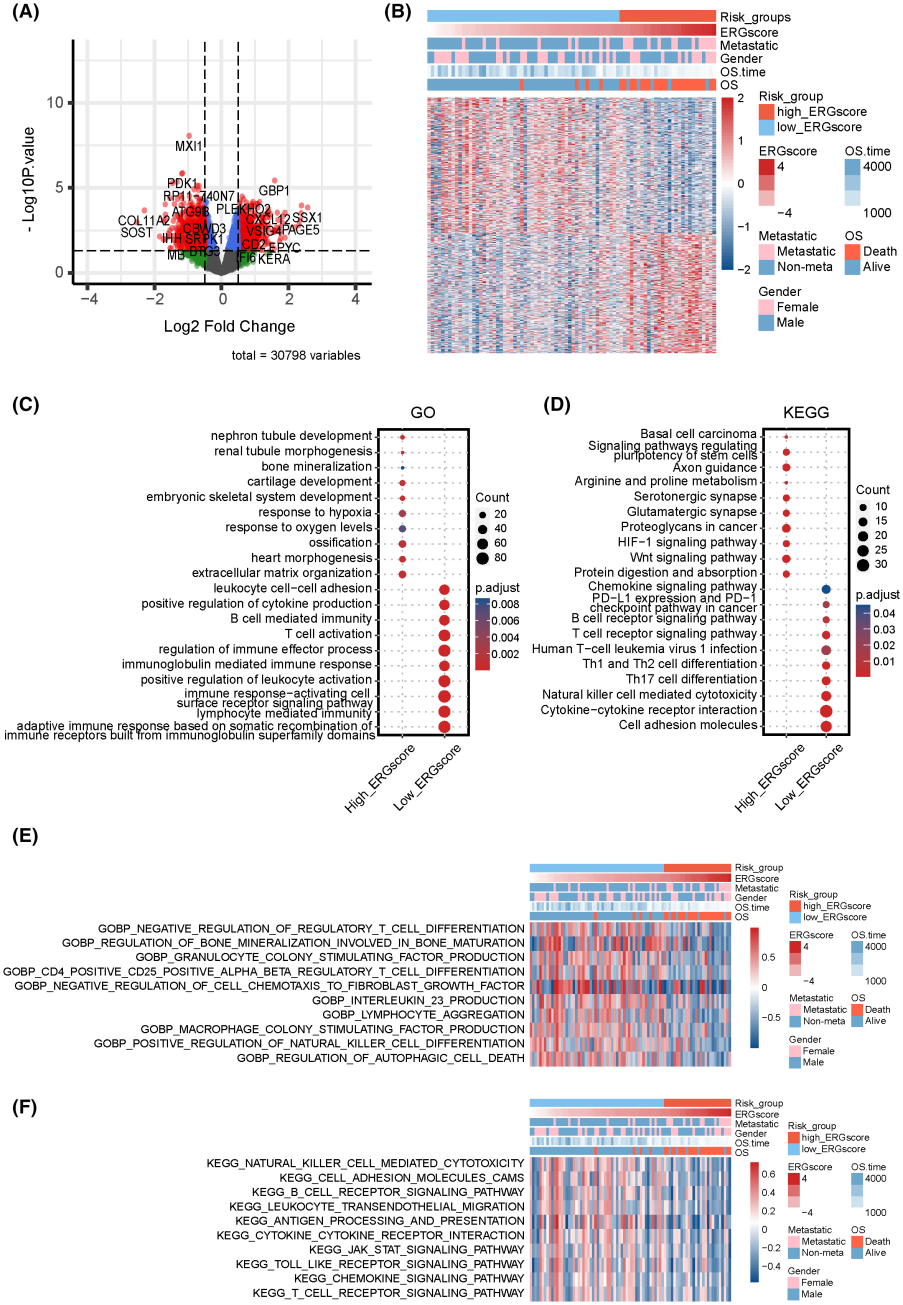
Functional enrichment characteristics based on the ERG signature stratification. (A) Volcano plot of DEGs between high‐ and low‐ERG‐score groups. Red plots represent statistically significant DEGs (|logFC| > 0.5 and adjust.*p* < 0.05). (B) Heatmap of statistically significant DEGs between high‐ and low‐ERG‐score groups. Pivotal items of GO (C) and KEGG (D) enrichment analyses based on the marker genes (logFC >0.5 and adjust.*p* < 0.05) in high‐ and low‐ERG‐score groups. Heatmaps of GSVA analysis for ERG signature subgroups based on GO‐biological‐process (E) and KEGG (F) gene sets in the MSigDB database.

We processed the expression profile to further investigate the molecular mechanisms related to ERG signature stratification. We screened out DEGs (|logFC| > 0.5 and adjusted *p* < 0.05) between the high‐ and low‐ERG‐score groups, which are shown in the volcano plot and heatmap (Figure 4A,B). Then GO and KEGG enrichment analyses were conducted to investigate the key signaling pathways and molecular functions. Based on these DEGs, the top 10 GO processes and KEGG pathways were displayed with “ggplot2” R package. GO biological process analysis showed that the high‐ERG group was mainly concentrated in hypoxia, cell stemness, and differentiation‐related processes, including cartilage development, ossification, and extracellular matrix organization. In contrast, the low‐ERG group was concentrated in immune processes, such as T‐cell activation, positive regulation of cytokine production, and positive regulation of leukocyte activation (Figure 4C). Regarding KEGG analysis, the high‐ERG group was enriched in tumor‐progress‐related pathways, including signaling pathways regulating the pluripotency of stem cells, the HIF‐1 signaling pathway, and the Wnt signaling pathway. However, the low‐ERG group was correlated with immune pathways like NK cell‐mediated cytotoxicity, Th17 cell differentiation, and T cell receptor signaling pathways. Additionally, GSVA analysis based on “c2.cp.kegg.v7.4.symbols” and “c5.go.bp.v7.4.symbols” gene sets were exhibited in heatmaps, abundances of immune‐related biological processes and pathways were statistically higher in the low‐ERG group (Figure 4E,F). These results indicate that ERG signature stratification correlates with tumor progression and OS immune microenvironments.

### 3.4 Single cell sequencing evaluated the correlation between ERG signature stratification and immunity

**FIGURE 5:**
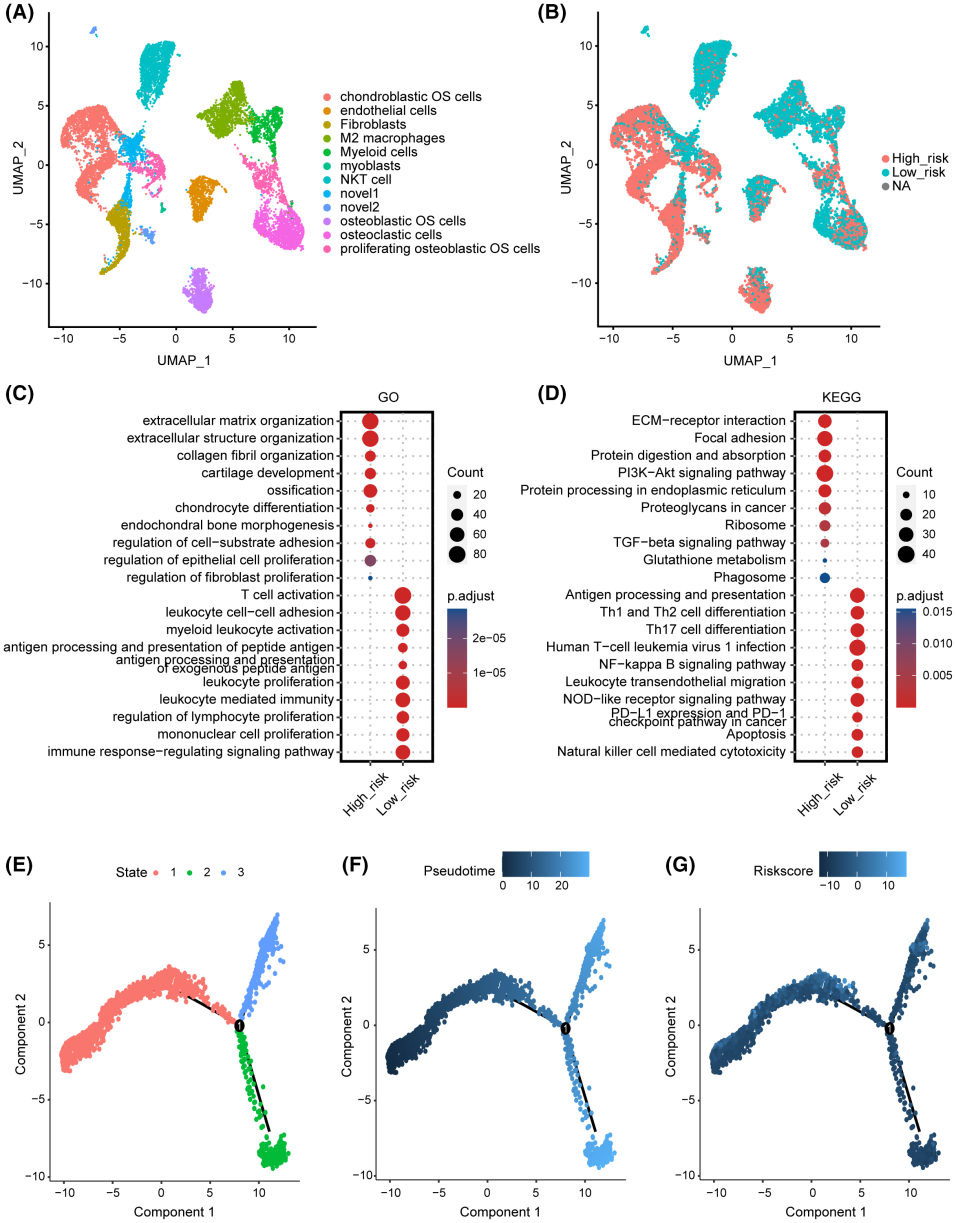
Single‐cell sequencing investigating the correlation between ERG signature stratification and the tumor microenvironment in OS. (A) UMAP visualization for 12‐cell annotation in OS single‐cell sequencing. (B) All cells were clustered into high‐ and low‐ERG‐score groups by ERG signature. NA represents that partial signature gene expression was not detected in the single‐cell sparse matrix. (C) GO enrichment analysis based on marker genes of two ERG score groups. (D) KEGG enrichment analyses based on marker genes of two ERG score groups. Color represents the adjust.*p* value, the darker the red, the higher the significance; the darker the blue, the lower the significance. (E) Pseudotime trajectory analysis divided OS cells into three different cell states. (F) Pseudotime pattern based on the pseudotime trajectory analysis on OS cells. (G) The ERG score distribution of OS cells on pseudotime trajectory analysis.

To further explore the potential correlation between the OS microenvironment and ERG signature, we extracted the scRNA‐seq profile of primary OS lesions to progress. All OS cells were clustered and annotated into 12 cell subclusters, including chondroblastic OS cells, endothelial cells, fibroblasts, M2 macrophages, myeloid cells, myoblasts, NK/T cells, osteoblastic OS cells, osteoclastic cells, and proliferating osteoblastic OS cells (Figure 5A). The ERG score of every cell was calculated using the ERG signature formula, and cells were divided into high‐ERG and low‐ERG groups by median value (Figure 5B). We observed that most chondroblastic OS cells, osteoblastic OS cells, proliferating osteoblastic OS cells, fibroblasts, and osteoclastic cells were marked as high‐ERG‐score. In contrast, NK/T cells, myeloid cells, endothelial cells, and myoblasts belonged to the low‐ERG group. Subsequently, the “FindMarkers” function was performed to identify marker genes in two ERG subgroups. GO enrichment and KEGG pathway analysis revealed that high ERG score cells were relevant to cancer‐progression‐related processes, including GO terms like extracellular matrix organization, ossification, and regulation of cell‐substrate adhesion, KEGG pathways‐like ECM‐receptor interaction, focal adhesion, PI3K‐Akt signaling pathway, and the TGF‐beta signaling pathway (Figure 5C,D). Low ERG score cells were concentrated in immune response processes, including GO terms like T cell activation, leukocyte cell–cell adhesion, and myeloid leukocyte activation, KEGG pathways like antigen processing and presentation, Th1 and Th2 cell differentiation, NF‐kappa B signaling pathway, NOD‐like receptor signaling pathway, PD‐L1 expression, and PD‐1 checkpoint pathway in cancer, apoptosis, and natural killer cell‐mediated cytotoxicity (Figure 5C,D). Furthermore, the pseudotime trajectory analysis was conducted based on OS cells, and three cell states were identified (Figure 5E). Notably, as the pseudotime increased, OS cells tended to have higher ERG scores (Figure 5F,G). Moreso, OS cells around branch point 1 were clustered into four subtypes, and the DEGs were identified (Figure S3A). The GO enrichment analysis was performed based on the DEGs of four subtypes (Figure S3B–E). The expression level of the signature genes in different cell states is displayed in Figure S4. These results are consistent with those in the TARGET patient cohort, in which ERG scores for cells or patients were positively related to cancer‐progression pathways and negatively correlated with immune response and cell death patterns.

### 3.5 Cell communication pattern of the ERG signature

**FIGURE 6:**
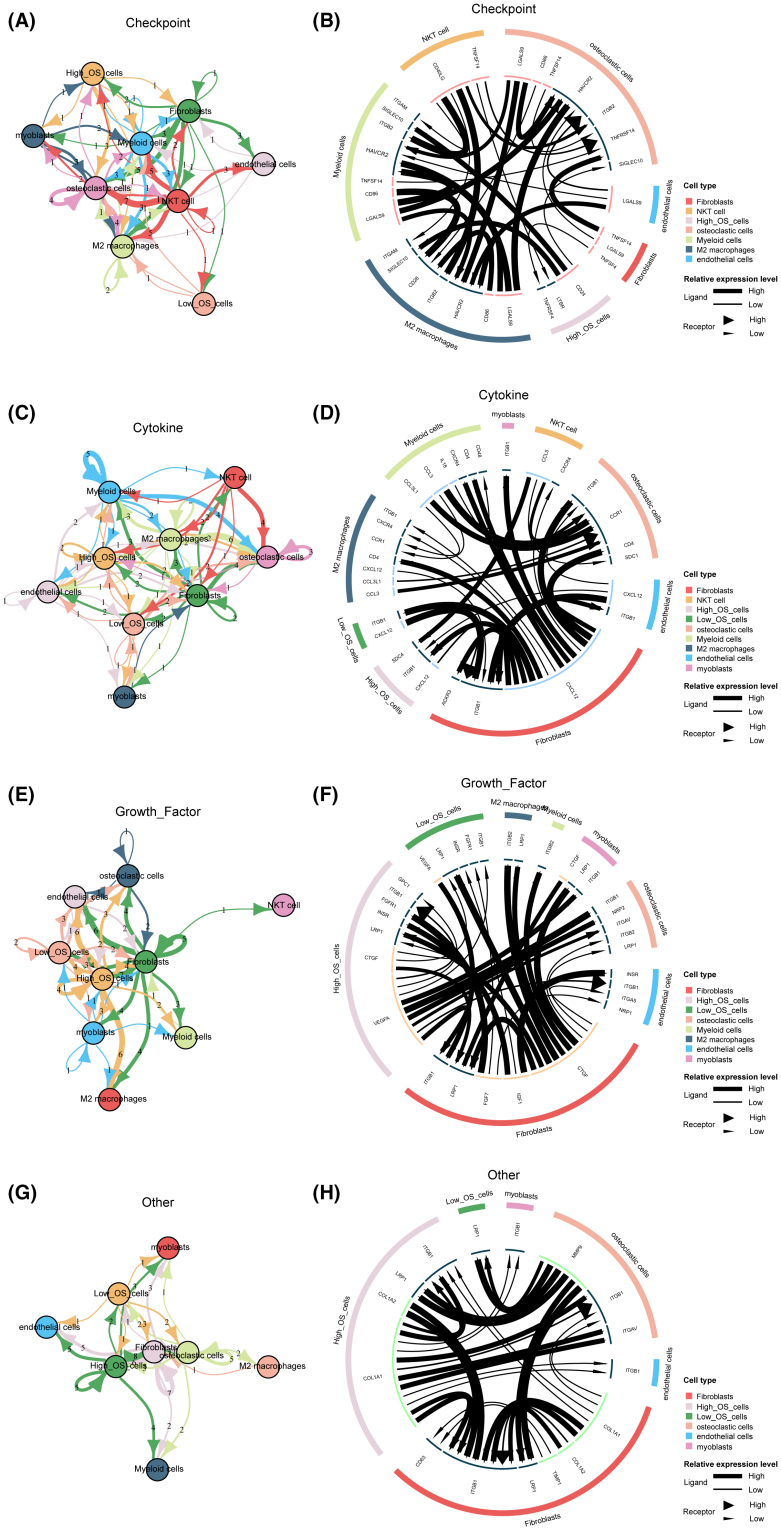
Cell communication pattern of the EMT‐related signature. (A) Top cellular signaling pathways based on checkpoints among microenvironment cells and two ERG score groups of OS cells. (B) Cell communication pattern based on checkpoints among microenvironment cells and two ERG score groups of OS cells. (C) Top cellular signaling pathways based on cytokines among microenvironment cells and two ERG score groups of OS cells. (D) Cell communication pattern based on cytokines among microenvironment cells and two ERG score groups of OS cells. (E) Top cellular signaling pathways based on growth factors among microenvironment cells and two ERG score groups of OS cells. (F) Cell communication pattern based on growth factors among microenvironment cells and two ERG score groups of OS cells. (G) Top cellular signaling pathways based on other ligands among microenvironment cells and two ERG score groups of OS cells. (H) Cell communication pattern based on other ligands among microenvironment cells and two ERG score groups of OScells.

Different intercellular signaling pathways regarding checkpoints between two ERG score subgroups of OS cells and microenvironment cells were investigated (Figure 6A,B). Notably, CD24, LTBR, and TNFRSF4 were the most activated signals in high‐ERG‐score OS cells. Different intercellular signaling pathways regarding cytokine between two ERG score subgroups of OS cells and microenvironment cells were also investigated (Figure 6C,D), in which SDC4, ITGB1, and CXCL12 were the most activated signals in high‐ERG‐score OS cells. Different intercellular signaling pathways regarding growth factors between two ERG score subgroups of OS cells and microenvironment cells were visualized in Figure 6E,F. These data demonstrated that VEGFA, CTGF, and LRP1 were the most activated signals in high‐ERG‐score OS cells. Different intercellular signaling pathways regarding other between two ERG score subgroups of OS cells and microenvironment cells were visualized (Figure 6G,H), and it was found that COL1A1, COL1A2, LRP1, and ITGB1 were the most activated signals in high‐ERG‐score OS cells.

### 3.6 Predictive performance of ERG signature on therapeutic strategies

**FIGURE 7:**
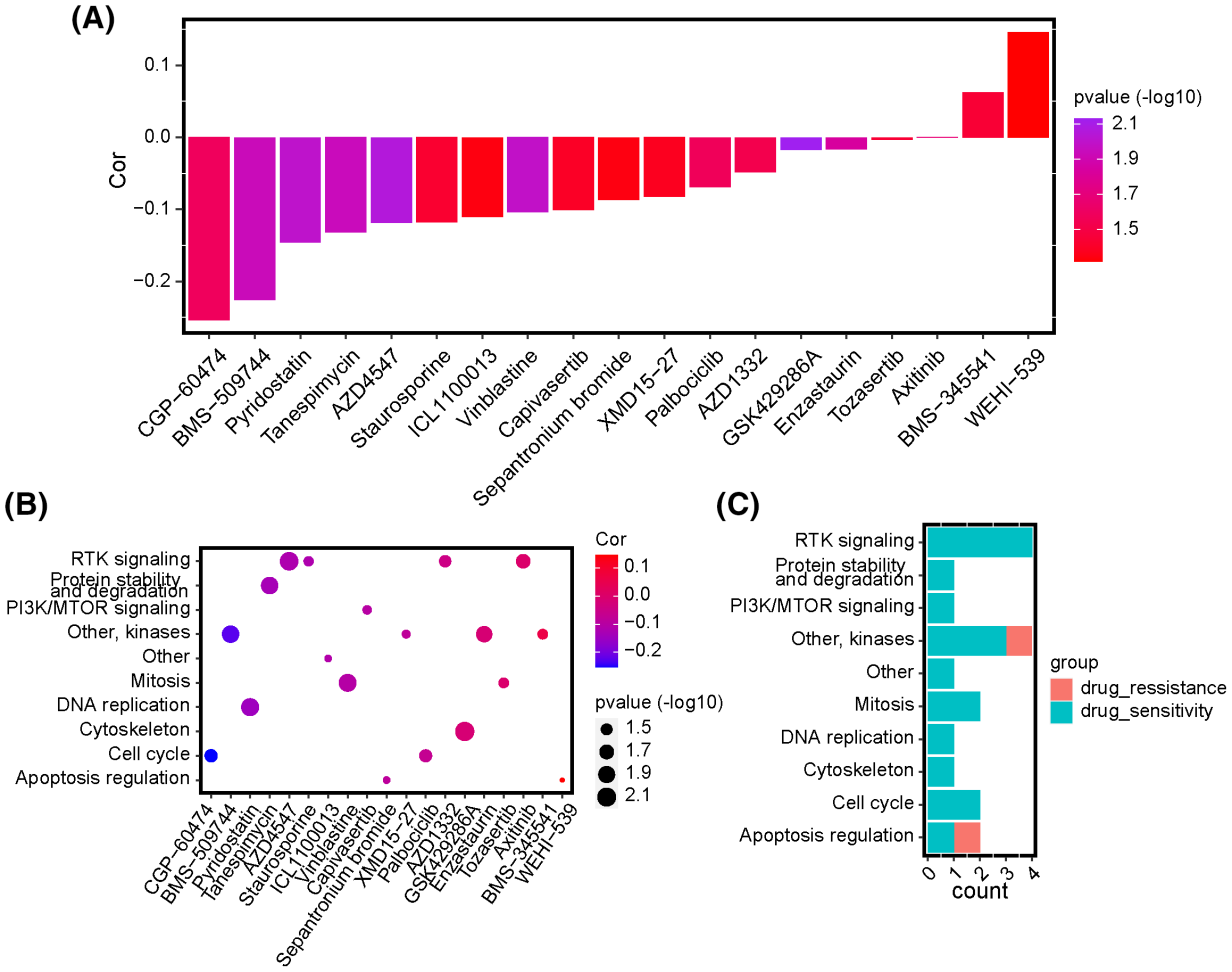
Correlations between the ERG signature and drug responses in GSDC pan‐cancer cell lines. (A) Bar diagram of the correlation between ERG score and IC50 of anticancer drugs in pan‐cancer. Altitude represents the correlation, the higher the altitude, the greater the correlation. Color represents statistical significance (*p*‐value). The more purple the color, the greater the significance. (B) Scatter diagram of the correlation between IC50 of anticancer drugs and targeting signaling pathways. The size of the plots represents statistical significance (*p*‐value). Red represents a positive correlation, and blue represents a negative correlation. (C) The bar diagram exhibits the numbers of sensitive and resistant drugs regarding the targeting pathways.

According to the Genomics of Drug Sensitivity in Cancer (GSDC) database, we used spearman analysis to evaluate the correlation between ERG scores and IC50 of drugs in cancer cell lines. Positive correlation represented enhanced drug resistance of cell lines. In contrast, negative correlation represented enhanced drug sensitivity. The results showed that higher ERG scores were associated with enhanced drug resistance of cancer cell lines to WEHI‐539 and BMS‐345541. Still, they promoted the drug sensitivity of cancer cell lines to multiple drugs, including CGP‐60474, BMS‐509744, pyridostatin, tanespimycin, AZD4547, staurosporine, and other drugs (Figure 7A). Targeted signaling pathways of the above drugs are displayed in the scattergram (Figure 7B,C). The histogram shows that drugs whose sensitivity correlated with increasing ERG score primarily targeted RTK signaling, kinases, mitosis, and cell cycle. Furthermore, drugs whose resistance correlated with increasing ERG score targeted apoptosis regulation and kinases.

### 3.7 Biological correlations of CDK3 in OS cells and Pan‐cancer

**FIGURE 8:**
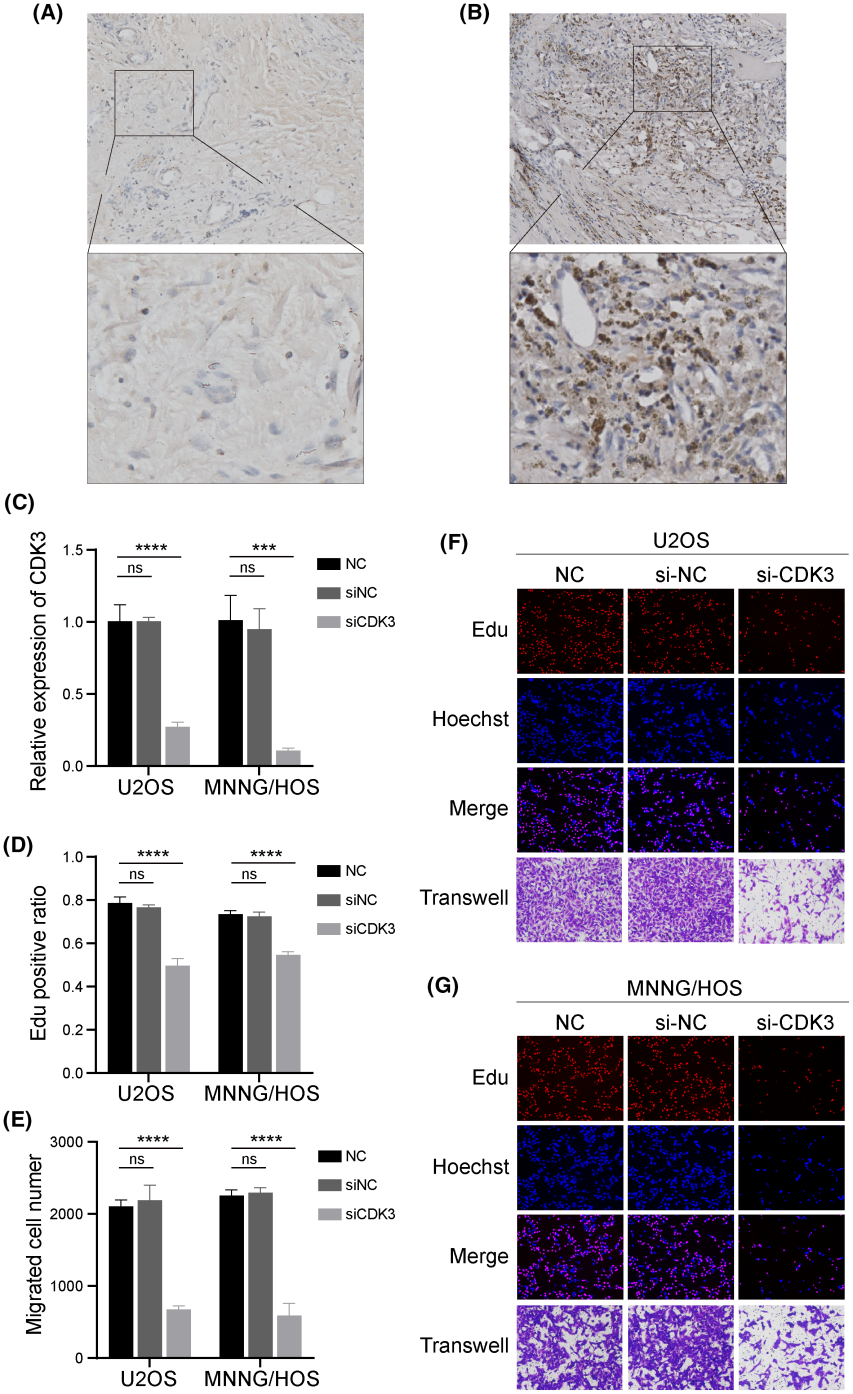
In vitro validation on CDK3. Representative immunohistochemical images of expressions of CDK3 in para‐carcinoma (A) and OS (B) tissues. (C) The relative expression level of CDK3 in normal control, siNC, and siCDK3 groups in U2OS and MNNG/HOS. (D) EdU‐positive ratios were calculated (*n* = 3) in normal control, siNC, and siCDK3 groups in U2OS and MNNG/HOS. (E) Migration cell numbers (*n* = 3) were counted after si‐RNA transfection. Representative images of EdU (red), Hoechst staining (blue), and transwell (purple) results in normal control, siNC, and siCDK3 groups in U2OS (F) and MNNG/HOS (G). Ordinary one‐way ANOVA test ***p < 0.001; ****p < 0.0001; ns, no significance.

Among the seven ERGs in our constructed prognostic signature, CDK3 is the most influential cancer‐promoting gene. It has the highest calculation coefficient in the formula, so we regarded CDK3 as the pivotal gene of the ERG signature for further investigation and verification. Based on IHC analysis, the expression of CDK3 was upregulated in OS tissue compared with paracancerous normal tissue (Figure 8A,B). We knocked down the expression of CDK3 using siRNA in two OS cell lines, U2OS and MNNG/HOS (Figure 8C). Following the knocked down, we observed that the Edu‐positive ratio and migration number of OS cells were significantly decreased in si‐CDK3 groups compared with normal control groups (Figure 8D–G). The proliferation and migration capabilities of OS cells were inhibited CDK3 expression was downregulated.

## 4 DISCUSSION

Treatment for OS patients requires the close cooperation of multidisciplinary teams. A thorough OS therapy must involve surgical removal of all detectable tumor lesions and preoperative plus postoperative polychemotherapy. However, there is a significant need to develop new approaches to improve the prognosis of OS patients with unresectable, metastatic, or recrudescent lesions.^1^, ^2^ Increasing studies concentrate on the role of EMT in predicting overall survival.^26^, ^27^ From these investigations, it has been reported that EMT development could result in drug resistance in lung cancer, breast cancer,^28^, ^29^, ^30^ and metastasis in breast cancer, OS, and bladder cancer,^31^, ^32^, ^33^ which may explain the contribution of EMT to a worse prognosis. Researchers have reported EMT as a critical event in OS metastasis,^34^ and many independent EMT‐related prognostic genes in OS have been explored.^35^, ^36^ However, EMT is a complicated biological process regulated by multiple genes. Integrating EMT‐related genes from transcriptional profiles might be significant to further understand the EMT process. Prognostic‐related gene signatures from sequencing data have been highlighted when identifying the risk stratification for cancer patients, predicting their survival, and determining individualized treatment.

This study constructed a novel seven‐gene signature based on EMT‐related genes, including CDK3, MYC, UHRF2, STC2, COL5A2, MMD, and EHMT2. The risk stratification regarding high and low ERG scores could precisely predict the overall survival of OS patients, which was demonstrated in two independent cohorts. When considering multiple clinical characteristics, a higher ERG score was significantly correlated with metastasis instead of other features like age or gender, which suggested the ERG signature might be a potential and reliable biomarker for OS metastasis. The ssGSEA and ESTIMATE methods were significant in investigating tumor immune microenvironments and predicting prognosis.^19^, ^21^ Our results suggest a higher abundance of immune cell expression and immune responses in the low‐ERG‐score group, consistent with dominant perceptions that more infiltration of immune‐activation cells was associated with better clinical outcomes of cancers.^37^ In further analyses on single‐cell sequencing, malignant OS cells, and fibroblasts were identified as high‐ERG‐score cells, while immune‐activation cells were low‐ERG‐score. Hence, we speculated that our ERG signature was involved in shaping the immune microenvironment of OS.

EMT is a sophisticated biological process involving multiple pathways. The function enrichment and GSVA analyses revealed that the low ERG score was mostly enriched in immune‐related pathways. In contrast, cell stemness‐related processes, the Wnt signaling pathway, and the HIF‐1 signaling pathway were significantly correlated with the high‐EMT‐score group. Our single‐cell method identified the enrichment of focal adhesion and ECM‐receptor interactions in cells with a high ERG score. Furthermore, accumulating studies have expanded on the significant role of EMT in the enrichment of cancer stemness and therapy resistance.^38^, ^39^ The Wnt signaling pathway activation could stimulate EMT by activating EMT‐related transcription factors, including Snail, Slug, Twist, ZEB1, and ZEB2.^40^ Additionally, activation of HIF‐1α in a hypoxic tumor microenvironment would affect the metabolism, angiogenesis, and survival of tumor cells and contribute to the development of EMT.^41^ Focal adhesion signaling is crucial in organizing the actin cytoskeleton and shaping cell motility, proliferation, and differentiation capabilities.^42^ The extracellular matrix (ECM) could regulate cell proliferation, migration, differentiation, and metabolism via integrin or interaction with cell surface receptors.^43^ Collectively, these results suggest the potential biological processes involved in our ERG signature built the groundwork for further mechanistic research. Importantly, our chemotherapy analysis suggested that drugs with sensitivity to high ERG scores mainly targeted RTK signaling, kinases, mitosis, and cell cycle pathways might provide a reference for individualized treatment for OS.

Our ERG signature identified five genes as risk factors, including CDK3, MYC, UHRF2, STC2, and COL5A2, while MMD and EHMT2 were protective factors. The correlation between EMT and risk factors evaluated in this study was previously investigated and reported. Lu et al. reported that CDK3 could promote EMT in colorectal cancer, conjugating AP‐1 activation by phosphorylating c‐Jun at Ser 63 and Ser 73.^44^ Notably, c‐Myc is overexpressed in multiple malignant tumors as an oncoprotein and a transcription factor. Chen et al. found that combining WDR5 and the MBIIIb motif of c‐Myc was essential to promote the EMT and metastasis of cholangiocarcinoma.^45^ Lai et al. also identified the upregulation of many EMT‐TFs in UHRF2‐overexpressing cells based on the analysis of proteome‐wide TF DNA binding activities.^46^ Moreso, Li et al. indicated that STC2 promoted cell EMT and glycolysis by activating ITGB2/FAK/SOX6 signaling pathway in nasopharyngeal carcinoma.^47^ Notably, COL5A2 was regarded as a mesenchymal marker. Importantly, the underlying connections among these ERGs are not clearly understood. Existing research has reported that multi‐targeted CDK inhibitors could restrain the activation of MYC in multiple myeloma cells^48^ and that MYC and UHRF2 were up‐regulated simultaneously in OS.^49^ Furthermore, well‐designed experiments are required to reveal the potential mechanisms and correlations among these genes. Remarkably, CDK3 was the most crucial gene for determining ERG score level by calculating the most significant coefficient among all seven genes. However, little research has been reported on the correlation between CDK3 and OS. Thus, we analyzed the CDK3 level to assess the role of ERG signatures.

CDK3 is a significant driver in retinoblastoma (Rb) phosphorylation during the G0/G1 transition of the cell cycle. The expression of CDK3 is deficient in normal tissue but overexpressed in many cancers.^50^ In our research, CDK3 was upregulated in OS tissue, and downregulating of CDK3 would inhibit the proliferation and migration capabilities of OS cells. Thus, we speculated that the overexpression of CDK3 in OS might correlate with mechanisms of proliferation and migration. Notably, CDK3 has potential utility as a therapeutic target and prognostic biomarker for OS. However, little research about the possible mechanisms between CDK3 and immunity has been reported, and further exploration of the mechanisms of CDK3 affecting tumor immunity and invasiveness is needed.

We recognize several limitations in our study that should be noted. First, our retrospective study was based on two public datasets in which the sample capacity of OS patients was small, and clinical characteristics were incomplete. More analyzable transcriptome data is yet to be explored for more accurate modeling, and more independent cohorts are necessary to validate the ERG signature. However, several studies have demonstrated the feasibility of using these two datasets we used to create risk models for OS.^49^, ^51^, ^52^ Thus, we believe that there is a considerable degree of reliability in our research. Second, though our evidence showed that the seven‐gene ERG signature had good performance in predicting OS prognosis, specific mechanisms of these genes in EMT progress and their crosstalk remain to be investigated. Third, our results suggested ERG signature correlated with immunosuppression in the OS microenvironment. However, further well‐designed and prospective mechanism experimental schemes are needed to verify our results.

## 5 CONCLUSION

In summary, EMT is a malignant progression correlated with a worse prognosis in OS patients. Here we constructed an ERG signature to predict the immune infiltration, tumor progression, and prognosis of OS patients, which might reference the traditional OS staging system and potentially facilitate individualized treatment. We also confirmed the cancer‐promoting function of CDK3 through proliferation and migration‐related experiments. However, further exploration is necessary to reveal the potential mechanism among these genes in OS and therapeutic efficacy.

## AUTHOR CONTRIBUTIONS

**Haoli Gong:** Conceptualization (equal); data curation (lead); formal analysis (equal); funding acquisition (lead); software (lead); supervision (supporting); validation (supporting); writing – original draft (lead); writing – review and editing (supporting). **Ye Tao:** Formal analysis (equal); methodology (supporting); validation (supporting); writing – original draft (supporting); writing – review and editing (supporting). **Sheng Xiao:** Data curation (supporting); formal analysis (supporting); methodology (supporting); writing – original draft (supporting); writing – review and editing (supporting). **Xin Li:** Investigation (equal); methodology (supporting). **Ke Fang:** Investigation (supporting); methodology (supporting). **Jie Wen:** Investigation (supporting); validation (supporting); writing – review and editing (supporting). **Ming Zeng:** Validation (supporting); writing – review and editing (supporting). **Yiheng Liu:** Funding acquisition (supporting); writing – review and editing (equal). **Yang Chen:** Conceptualization (equal); formal analysis (equal); methodology (supporting); supervision (lead); validation (equal); visualization (equal); writing – original draft (supporting).

## FUNDING INFORMATION

This work was supported by Natural Science Foundation of Hunan Province (2022JJ40218), Young Doctors Fund of Hunan Provincial People's Hospital (BSJJ202118).

## CONFLICT OF INTEREST STATEMENT

The authors declare that the research was conducted without any commercial or financial conflict of interest.

## ETHICS STATEMENT

The studies involving human participants were approved by the institutional review board (IRB) of the Hunan Provincial People's Hospital (No: 2021‐S104). Written informed consent for participation was not required.

## ETHICS APPROVAL AND CONSENT TO PARTICIPATE

Not applicable.

## CONSENT FOR PUBLICATION

Not applicable.

## Supporting information


Figure S1.

Figure S2.

Figure S3.

Figure S4.

Table S1.

Table S2.

Table S3.

## Data Availability

The original datasets/matrixes are available in the GEO repository (https://www.ncbi.nlm.nih.gov/geo/), UCSC Xena database (https://xena.ucsc.edu/), Epithelial‐Mesenchymal Transition Gene Database (http://dbemt.bioinfo‐minzhao.org/download.cgi), the Molecular Signatures Database v7.5.1 (https://www.gsea‐msigdb.org/gsea/msigdb/index.jsp), and GDSC database (http://www.cancerrxgene.org/).
