# Supplementary figures and images for: Identification of an EMT‐related gene‐based prognostic signature in osteosarcoma
Source: Cancer Med. 2023 Apr 27;12(11):12912–28. doi: 10.1002/cam4.5942 (PMC10278480; doi:10.1002/cam4.5942)

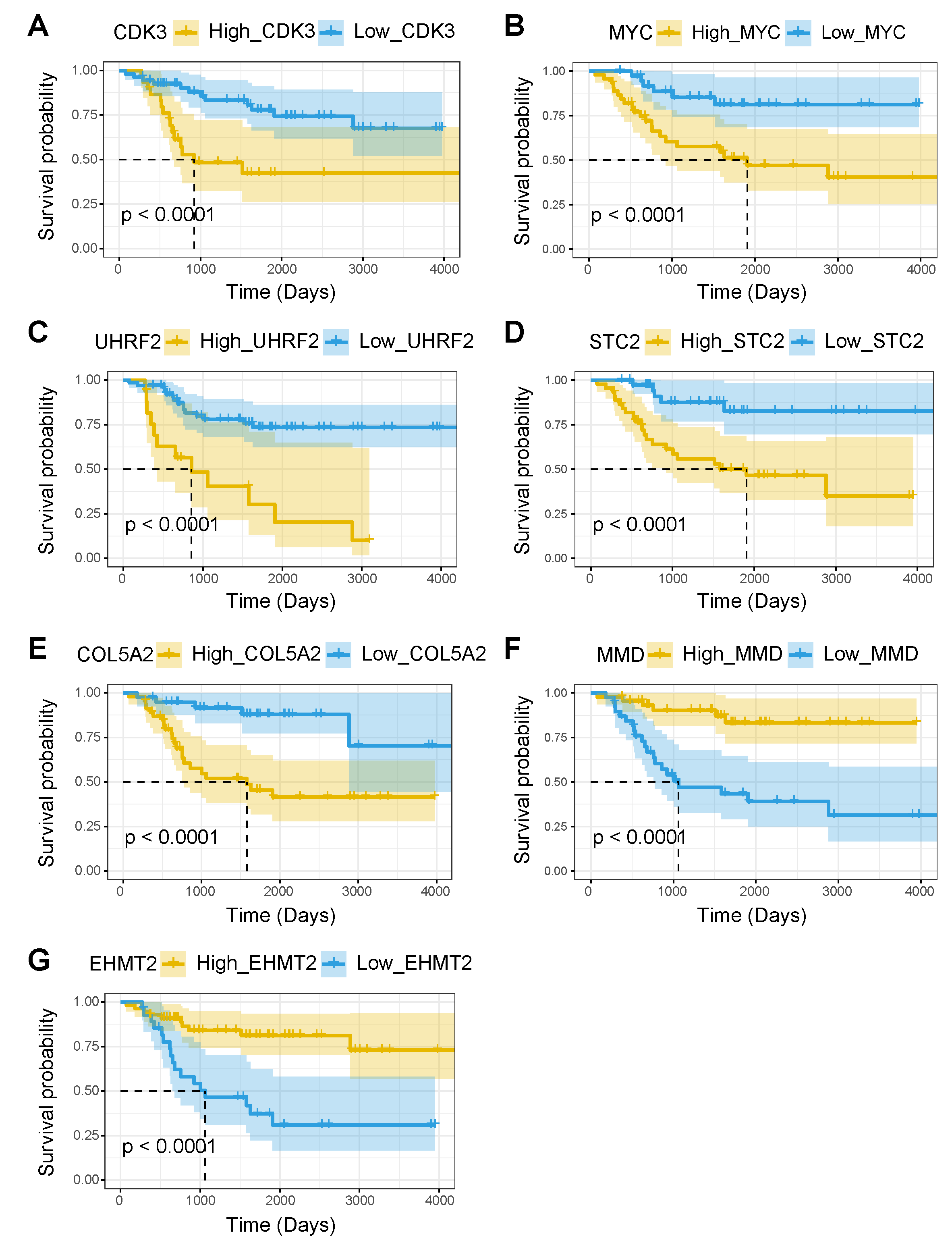

Supplement: Supplementary file 1 — Figure S1. [file CAM4-12-12912-s007.tif]

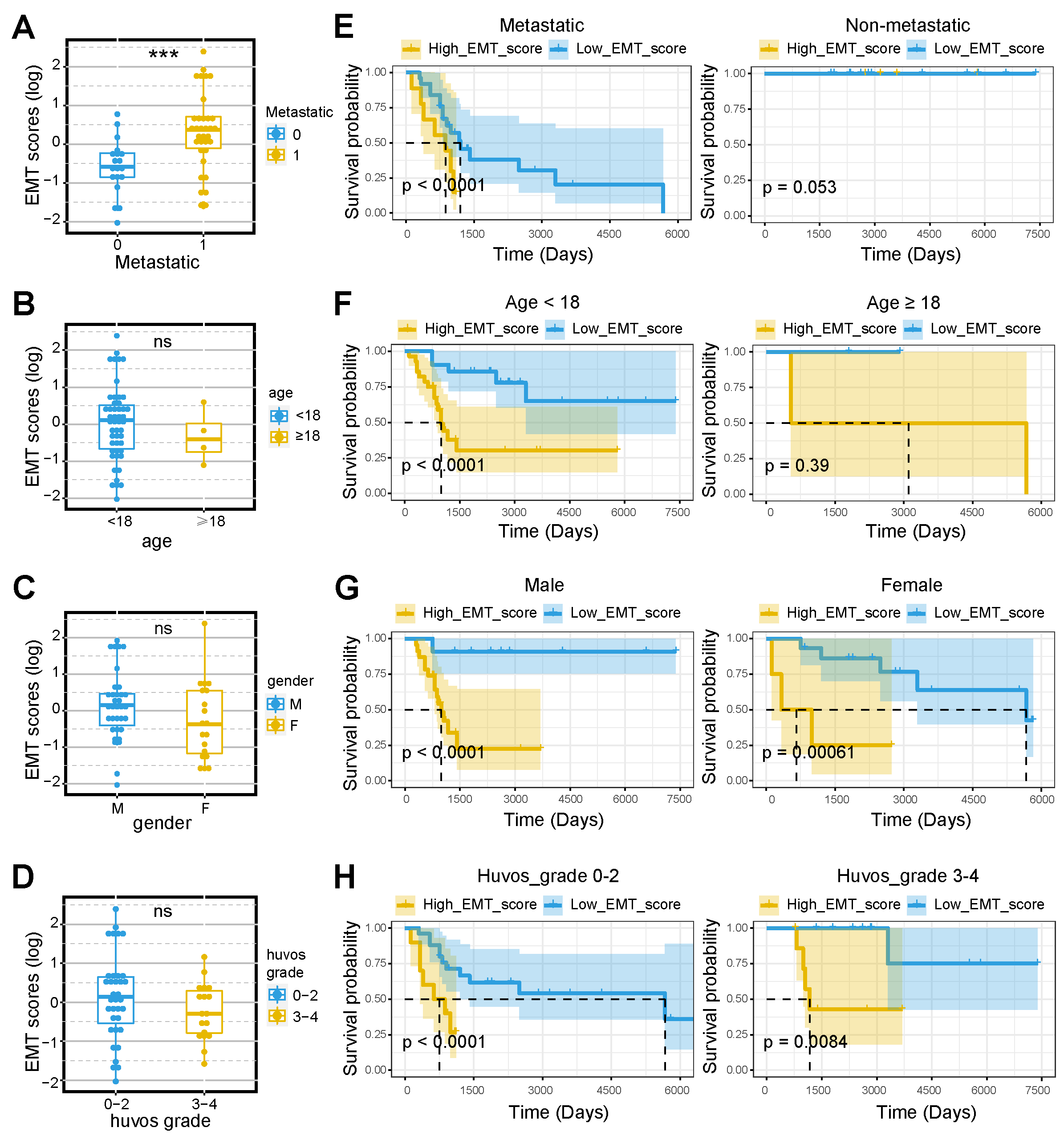

Supplement: Supplementary file 2 — Figure S2. [file CAM4-12-12912-s003.tif]

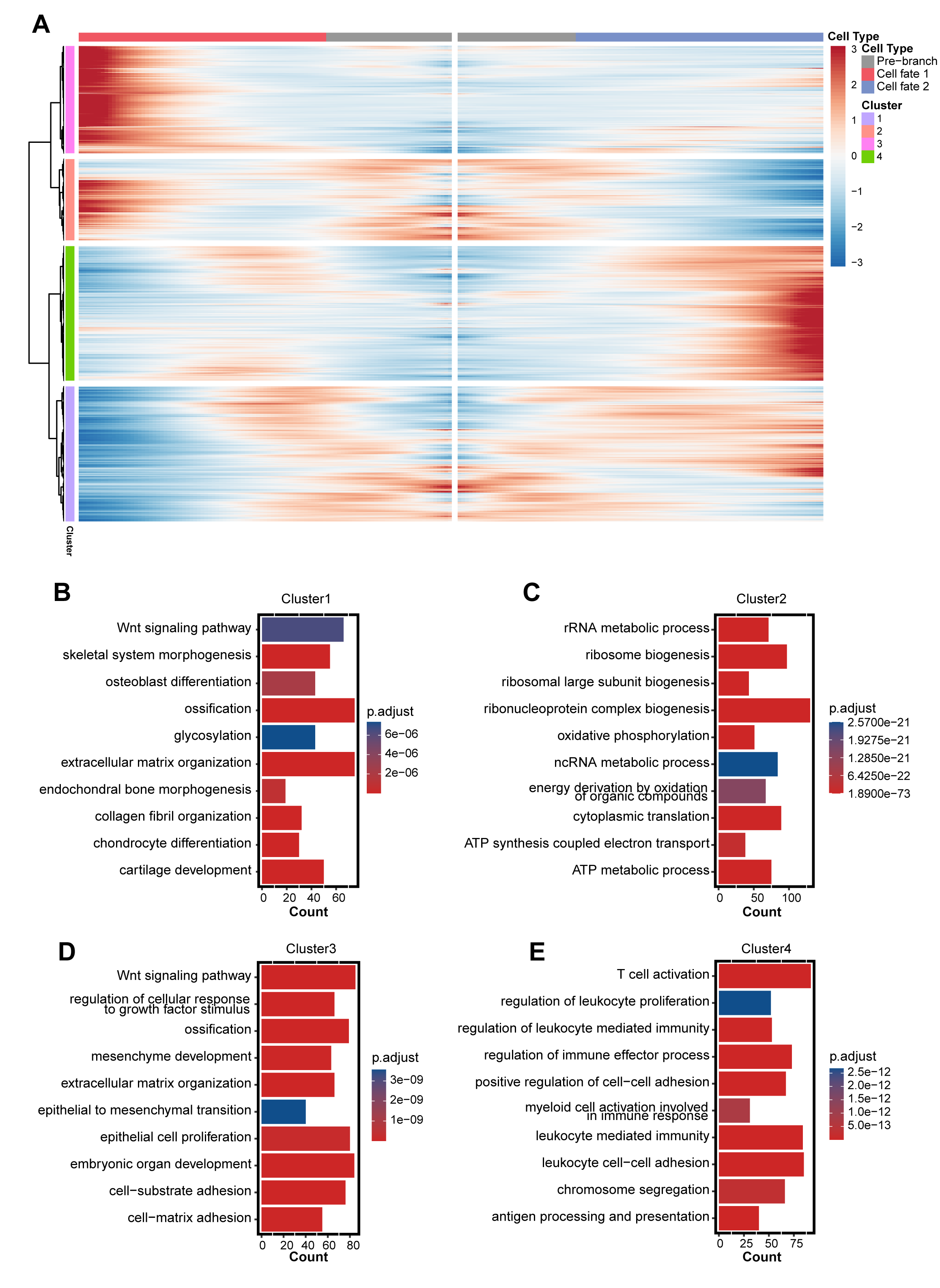

Supplement: Supplementary file 3 — Figure S3. [file CAM4-12-12912-s002.tif]

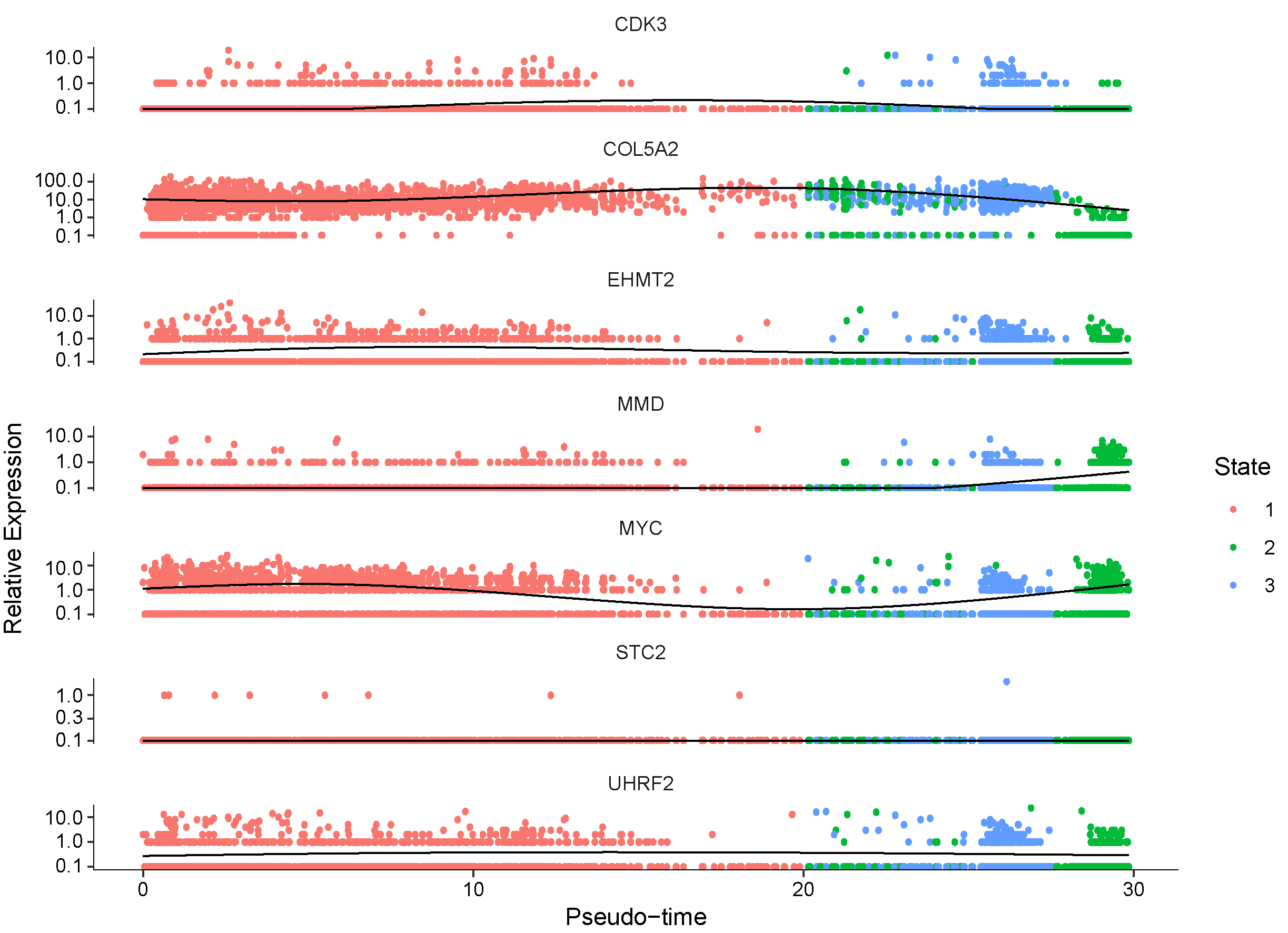

Supplement: Supplementary file 4 — Figure S4. [file CAM4-12-12912-s001.tif]
